# Catenary Electromagnetics for Ultra‐Broadband Lightweight Absorbers and Large‐Scale Flat Antennas

**DOI:** 10.1002/advs.201801691

**Published:** 2019-02-01

**Authors:** Yijia Huang, Jun Luo, Mingbo Pu, Yinghui Guo, Zeyu Zhao, Xiaoliang Ma, Xiong Li, Xiangang Luo

**Affiliations:** ^1^ State Key Laboratory of Optical Technologies on Nano‐Fabrication and Micro‐Engineering Institute of Optics and Electronics Chinese Academy of Sciences Chengdu 610209 China; ^2^ School of Optoelectronics University of Chinese Academy of Sciences Beijing 100049 China

**Keywords:** catenary optics, dispersion engineering, flexible, large‐scale fabrication, metasurface waves

## Abstract

Catenary functions are exciting and fundamental building blocks in constructing various kinds of waves in subwavelength structures. Here, a simple yet powerful approach inspired by catenary optics is proposed to realize efficient manipulation of electromagnetic waves in terms of both amplitude and phase. By properly engineering the catenary electromagnetic fields and frequency dispersion, lightweight metafilm‐based broadband absorbers with polarization‐independent bandwidth covering 0.65–6.2 GHz are experimentally achieved, and the bandwidth is further broadened to 0.9–40 GHz. With the same approach, a large‐scale flat antenna based on generalized reflection is demonstrated in the satellite communication system. To enable the batch manufacturing, a flexible substrate–based microfabrication process is developed with a minimum feature size of down to sub‐micrometer and total size up to almost 1 m. These results may provide important guidance for the design of metasurface‐based devices.

## 1 Introduction

Metasurfaces are 2D equivalents of metamaterials that are composed of subwavelength unit cells in an ultrathin surface.^1^, ^2^, ^3^, ^4^ By interacting with local electromagnetic field, according to the generalized Snell's law,^1^, ^5^, ^6^ metasurface‐based full control of light can be achieved in terms of amplitude, phase, dispersion, momentum, and polarization.^1^, ^2^, ^3^, ^4^ Owing to their versatile functionality, ultrathin feature, and ease of integration, these newly proposed surfaces have drawn enormous attentions. To date, many novel phenomena and devices have been proposed and realized such as negative refractive index,^6^ flat lenses,^7^, ^8^ invisibility cloaks,^9^ catenary optical devices,^10^ and holograms,^11^ among others.^12^, ^13^ However, the interactions between metasurface and the electromagnetic field are so complex, and it seems impossible to give a concise yet accurate description. As a generalized solution to quantitatively describe the light–matter interactions in subwavelength scale has not been concluded, the prediction of an unknown metasurface is mainly based on iteratively solving Maxwell's equations with the finite‐element modeling or finite‐difference time‐domain method. So far, the design of a metasurface with certain functionalities is performed in a bottom‐up way that relies on the expertise of the designer to optimize the unit cells in processes of trial and error, which is time wasting and often leads to locally optimized solutions. Thus, obtaining the accurate linkage between the structural profile and its responses is the key to realize inverse design with maximum efficiency.

As the constituent, functionality, and operation band of metasurfaces differ greatly from each other, the conclusion of a generalized solution once and for all is still far from perfect. However, for some specific cases, it is possible to bridge the linkage between metasurface and its response spectrum. The well‐known example is the case of 1D photonic crystals (they can be treated as unpatterned metasurfaces) that their optical responses can be quantitatively calculated by the transfer matrix method.^14^, ^15^ As a result, many optimization methods for these structures are proposed and the robustness of this kind of structures shows great advances over their patterned counterparts.^16^, ^17^, ^18^ Although previous works demonstrated that some patterned metasurfaces can be well described by a mathematical model, the conclusion is drawn by retrieval methods that cannot be employed for inverse designs directly.^19^

Recently, it was demonstrated that the field distribution and frequency dispersion of metasurface waves in subwavelength 1D metallic gaps can be well characterized by a catenary model.^20^, ^21^ Inspired by this, here we proposed a high‐accuracy mathematical model to describe catenary electromagnetic fields in both 1D and 2D structures, and to guide the design of functional metasurface devices. Meanwhile, a microfabrication process based on step exposure technique is proposed for precision fabrication on flexible substrates. Based on these innovations, lightweight broadband metasurface absorbers covering almost the entire useful microwave frequencies are experimentally demonstrated and a flat film antenna with performance comparable to its parabolic counterpart is also realized. We believe the design method as well as the fabrication process may open a new door for efficient design and large‐scale fabrication for the next‐generation functional metasurfaces.

## 2 Design and Methods

### 2.1 Revisiting of Catenary Electromagnetic Field Theory

**Figure 1:**
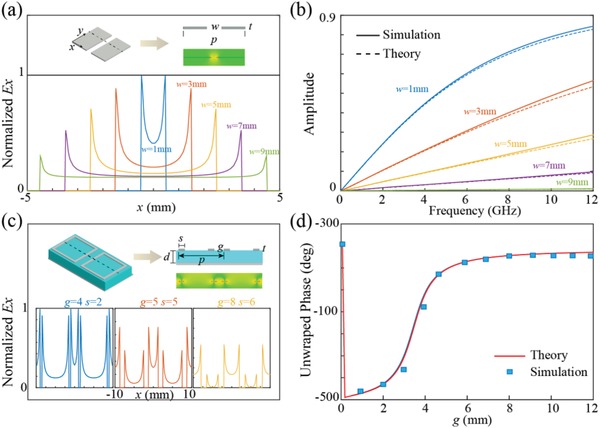
The catenary models in 1D and 2D structures. a) The extracted electric field distribution in metallic slit with various *w* at 10 GHz. The upper inset is the schematic of the subwavelength slit with *p* = 10 mm and the electric field of the incident wave is parallel to the *x*‐axis. b) The comparison of transmission amplitude between simulated results obtained by CST (solid lines) and their theoretical counterparts calculated by catenary model (dashed lines). c) The extracted electric field distribution in two adjacent CRRs with various *g* and *s*. The upper inset is the schematic diagram of the structure with *p* = 20 mm, *d* = 5 mm. d) The comparison of unwrapped phase between simulation and theory with different *g*. The working frequency is *f* = 4 GHz and *s* is fixed at 1 mm. Both results show great agreement even *g* approaches zero.

For the simplest case of 1D metallic slits shown in **Figure** **1**a under the illumination of transverse magnetic (TM) plane wave (the definition of TM wave is given in the caption of Figure 1), the electric field distributions mainly rely on the gap width *w* of the slit when the thickness of the structure *t* is in subwavelength scale (*t* ≪ λ). Figure 1a shows the *w* dependence of the electric field distributions along the dotted line in the inset. In our previous researches about meta‐surface‐waves,^20^, ^21^ we showed that the field distributions between the slits can be described by catenary functions as a result of the evanescent coupling. However, this assumption is only confirmed at certain conditions and rigorous demonstrations considering that the inner physics are not presented. To further confirm this issue in a generalized way, a detailed deduction based on Schwartz conformal transformation is carried out that manifests the distribution of electric field *E_x_* between the slits share a nearly perfect catenary form (details are given in Parts S1 and S2 in the Supporting Information).^22^ More interestingly, the effective impedance *Z*_eff_ of the surface also possesses similar catenary shape as follows

(1) $$Z_{\mathrm{eff}}=\frac{1}{4iF}$$

where *F* is a typical catenary model that can be expressed as

(2) $$F\left(p,w\right)=\frac{p}{\lambda }ln\;\left(\csc\left(\frac{\pi w}{2p}\right)\right)$$

where λ and *p* are the operation wavelength and period of the structure, respectively. Equations (1) and (2) give a direct linkage between the geometry of the slit and its corresponding electromagnetic response. The calculated and numerical simulated transmission amplitudes with various *w* are depicted in Figure 1b with nearly perfect agreement. As the catenary field distribution *E_x_* and the catenary model *F* own the same mathematical form and are both reversely proportional to *w*, we conclude that it is the catenary electric field distributions as well as the catenary frequency dispersions that lead to the catenary‐formed mathematical models in equivalent circuit theory.^23^

Although the aforementioned 1D structures are widely used in various metasurfaces, owing to their constrained degrees of freedom, the applications for these polarization‐dependent unit cells are still limited. To shed light on a more generalized 2D case, here, we employed the closed ring resonator (CRR) (shown in Figure 1c), which is one of the representative designs for polarization‐independent metasurfaces, as an example. To broaden the catenary electromagnetics to a 2D version, we made similar analysis for CRR as mentioned above. Different from the case for metallic slits, we employed a reflective profile to analyze CRR which is consistent with the design of the broadband absorber and film‐based antenna. The catenary field distributions in CRRs can be divided to two parts: one is between the two arms of a resonator and the other is between adjacent resonators. The retrieved field profiles for both cases are shown in Figure 1c, which indicate that at fixed period *p* there are two variables (*g* and *s*) that can alter the distribution of the overall electromagnetic field. By making an analogy with the 1D case, the retrieved effective impedance of a given CRR can be obtained that also relies on the catenary function *F* (details are shown in Part S3 in the Supporting Information).^23^ To further confirm the validation of this catenary model, the simulated and calculated phase responses with various *g* at 4 GHz are depicted in Figure 1d. The simulated results matched their calculated counterparts perfectly even when *g* approaches zero. The aforementioned analysis suggests that by properly engineering the catenary electromagnetics in metasurface, arbitrary responses can be realized.

## 3 Results and Discussions

### 3.1 The Design and Characterization of the Broadband Absorber

**Figure 2:**
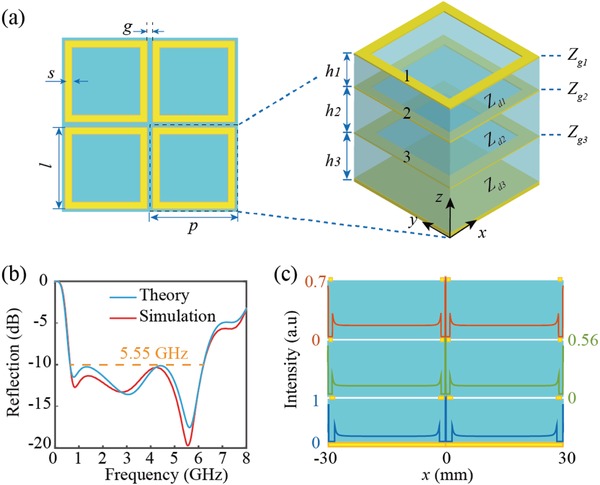
The design of the broadband absorber. a) The schematic of the absorber. Based on the catenary model, the response of this structure can be calculated directly that the parameters of this structure can be optimized to realize efficient absorption. The optimized parameters are *p* = 30 mm, *g* = 0.2 mm, *s*_1_ = *s*_2_ = 1 mm, *s*_3_ = 1.5 mm. *h*_1_ = 13 mm, *h*_2_ = *h*_3_ = 18 mm. *R_s_*_1_ = 30 Ω, *R_s_*_2_ = *R_s_*_3_ = 22 Ω. The dielectric is set as air with ε = 1 and the metal is nichrome with conductivity σ = 2.2 × 10^5^ S m^−1^. *Z_gi_*, *Z_di_* (*i* = 1, 2, or 3) are the effective impedance of the metasurface and the impedance of dielectric. b) Simulated and theoretical reflection from 0 to 8 GHz. The −10 dB bandwidth of our structure is 5.55 GHz with more than three octave bandwidths. c) The extracted electric field distribution along the trilayered metasurface at 5.56 GHz. As the distance between adjacent resonators is much smaller than that between two arms of a resonator, the intensity of electric field is much stronger in the former case.

With the development of modern society, perfect absorption plays a key role in sensing, communication, and stealth technology. Traditionally, efficient microwave absorption is achieved by ferrite‐based absorbing coatings.^24^, ^25^ However, the densities of these materials are quite large and they can hardly realize broadband absorption especially at ultralow frequencies, which hindered their applications in aviation and space fields. To overcome this challenge, we employed the aforementioned catenary model to design a broadband absorber with ultralow density. The schematic of the proposed absorber is shown in **Figure** **2**a that works from the P band to C band. The geometry of our device consists of trilayered CRR metasurfaces and a reflective ground plane that separated from each other by three dielectric layers (air) with distinctive thicknesses. By employing the generalized Fresnel's equations and transmission line theory (details are given in Part S4 in the Supporting Information),^3^, ^26^, ^27^ the reflection coefficient *r* of this structure can be well calculated as a function of *r*(*p*,*g*,*s*,λ,*d*), and the absorption can be easily obtained by *A* = 1 − |*r*|^2^ as the reflective ground plane can prevent transmission. Traditionally, the optimization of this kind of structures is mainly based on parameter scanning and results comparison. Such a design process is innately suffered from human‐guided errors, and the solution is often locally optimized. However, as the direct linkage between the structure and its performance can be well described by the catenary model, an automatic design process can be employed. For our device, the optimization process is akin to the case in our previous work for photonic crystals.^17^ Since the effective impedance of the metasurface can be accurately expressed with the catenary functions, it can be well treated as an effective sheet and, thus, the structure can be seen as a generalized photonic crystal. With the help of the Genetic Algorithm (GA), the related parameters can be identified automatically after several iterations to approach the desired absorption spectrum. The design principle for a desired spectrum based on GA is given in our previous work in detail.^17^ The optimized parameters as well as the involved materials are given in the caption of Figure 2a where *R*_s_ is the resistance of the metal film that can be calculated by *R*_s_ = 1/*σt* (σ is the conductivity of the metal and *t* is the corresponding thickness). The calculated absorption performance under normal incidence is depicted in Figure 2b. It should be noted that in order to make this structure in subwavelength scale and reduce the complexity in optimization, the period of the metasurfaces is fixed at *p* = 30 mm. To confirm the validity of our theoretical results, full wave simulation is carried out with the commercial software CST Microwave Studio (details are given in the Experimental Section). As shown in Figure 2b, both the theoretical and simulated results matched each other quite well and indicate that the −10 dB polarization‐independent reflection is realized from 0.65 to 6.2 GHz by this trilayered metasurface.

To evaluate the performance of our device comparatively, the theoretical limit under normal incidence, the Rozanov limit, for these nonmagnetic absorbers is calculated based on Equation (3), 28

(3) $$d_{l}=\left|\int_{0}^{\infty }\ln\left|R(\lambda )\right|d\lambda \right|/2\pi^{2}$$

where *d*_l_ is the total thickness for an ideal device. In our case, the theoretical value of *d*_l_ with the same performance in Figure 2b is 43.8 mm which is very close to the thickness of our device (*d*_t_ = 49 mm). The deviation ratio (defined as (*d*_t_ − *d*_l_)/*d*_t_) is about 10%, which is much smaller than its previous counterparts based on other design principles.^29^, ^30^ To further confirm the existence of catenary field, the extracted electric field distributions at each metasurface at 5.56 GHz is given in Figure 2c. Apparently, the distributions of these fields are different that indicate the effective impedances of these layers are altered to meet the requirement of an ideal absorber.

As the metasurfaces in our simulation are set as surfaces with no physical thicknesses, to meet the requirement in fabrication, the traditional printed circuit board (PCB) process is no longer valid because the thicknesses of boards are in millimeter scale. In order to realize deep‐subwavelength thickness of the structure, the CRRs must be printed on a dielectric film thinner than tens of micrometers. In our case, 50 µm polymide films were employed to serve as the substrate for the metasurfaces and the thicknesses of nichrome for the trilayered metasurface are varied from 100 to 200 nm to meet the desired resistance *R*. In fact, the precise fabrication for subwavelength patterns on flexible substrate is quite challenging, especially for large‐scale devices. Although some processes such as laser processing and film printing have been proposed, these techniques are not suitable for our case owing to their low fabrication precisions and small operation area. Here, we proposed a process that can enable batch manufacturing with high precision based on step exposure techniques (details are given in the “Experimental Section”).

**Figure 3:**
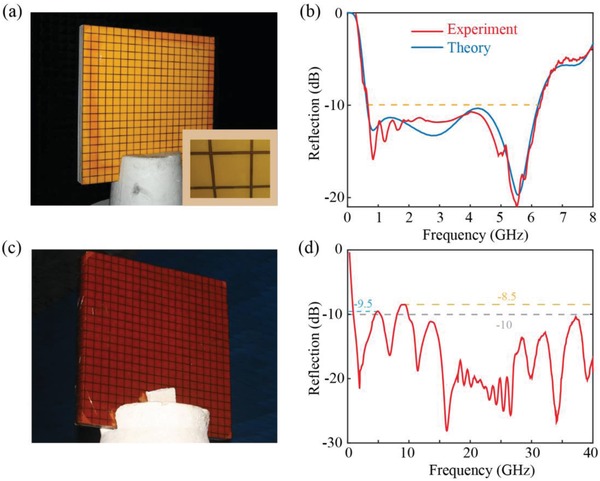
The fabricated samples and their measured results. a,b) The image of the foam‐based absorber and its performance from 0 to 8 GHz. c,d) The image of the absorbing honeycomb‐structure‐based material and its measured reflection from 0.3 to 40 GHz. Owing to the limitations of our testing equipment, the performance at higher frequencies cannot be measured.

After the metasurfaces are fabricated, for ease of characterization, polymethacrylimide (PMI) foam is employed in the experiment instead of air to serve as the dielectric layer. As the permittivity of PMI is only 1.05, such replacement has little influence on the response of the device (the comparison is plotted in Figure S5 in the Supporting Information). The image of the sample is depicted in **Figure** **3**a and the measured results are shown in Figure 3b accompanied with the simulated counterparts. It can be inferred from Figure 3a that the subwavelength structures are well patterned on the film with excellent uniformity that demonstrates the validity of our fabrication method. The measured results matched the simulated ones quite well with nearly the same −10 dB bandwidth. As there is no obvious discontinuity occurred in the measured region, the consistency of the results further confirmed the validity of our measurement. The slight discrepancy between measurement and simulation can be attributed to two reasons. First, the fabrication error and the roughness of the film cannot be fully removed in our process, which unavoidably leads to some deviations. Second, epoxy glue for adhering the film and PMI in the experiment is not considered in the simulation, which can also modify the response spectrum of our device. To further confirm the lightweight property of our device, we measured a 600 mm × 600 mm × 49 mm sample with a total quality of 1.09 kg. The corresponding density of our device is only 0.06 g cm^−3^ which is much lighter than traditional PCB boards and ferrite‐based coatings. In fact, the density of this device can be further decreased if we replace the PMI layers with air. In that case, the absorber will be a truly flexible structure that can be employed for other conformal applications.

Besides, the bandwidth of this kind of absorber can be further broadened if absorbing dielectric layers are employed. To demonstrate this issue, we replaced the bottom PMI with commercial honeycomb‐structured absorbing material (TGFW‐180, ARAMICORE). In this case, the structural parameters for the trilayered metasurface and the thicknesses of the dielectric layers are the same as the foam‐based device. The fabricated sample and corresponding measured results from 0.3 to 40 GHz are shown in Figure 3c,d. The operation bandwidth of this device shows a slightly blueshift compared with the former one that the −10 dB reflection bandwidth ranges from 0.9 to 40 GHz except for two narrow bands near 4.9 GHz (−9.5 dB) and 9 GHz (−8.5 dB). This can be attributed to the impedance mismatch of the whole structure with air. Owing to the limitations of our testing equipment, the performance at higher frequencies cannot be measured. It should be noted that as the permittivity and permeability of the absorbing material are unknown, the approximated simulation as shown in Figure S6 (Supporting Information) is performed by fitting the material parameters from its reflectance. Although this simulation result is not precise enough, it demonstrates that the combination of the catenary model with absorbing material can further expand the operation bandwidth of the device. In fact, if the parameters of the absorbing material are available, the performance of this structure can be further improved by our optimization method.

### 3.2 Characterization of the Film‐Based Antenna

**Figure 4:**
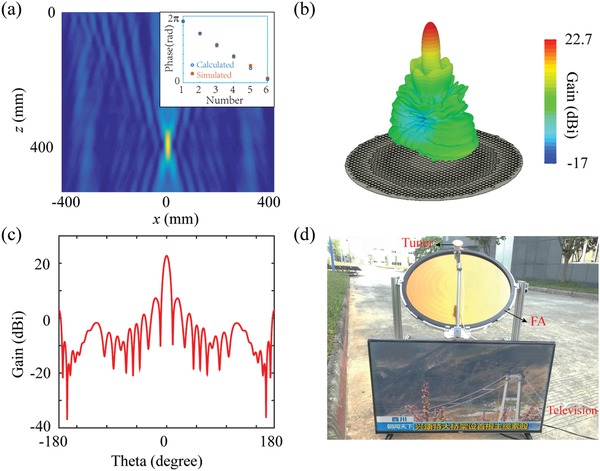
a) The simulated performance of our film antenna with 800 mm diameter and 400 mm focal length at 12 GHz. The inset depicts the phase responses of the six‐leveled unit cells in the antenna with different structural parameters obtained by the catenary model (blue) and numerical simulation (orange), respectively. b,c) The simulated results of 3D and 2D gain patterns at 12 GHz for a smaller antenna with 200 mm diameter and 100 mm focal length. d) The image of the satellite television system. In this system, the metafilm‐based antenna is responsible for focusing the signals emitted from ChinaSat 9 to the tuner. As this photo was taken outdoors, the strong sunlight influenced the contrast of the picture on the television.

In the aforementioned case, we only employed the catenary model to manipulate the amplitude of the incident wave. In fact, this model could be used to predict the phase response of the unit cell as well (as shown in Figure 1d). Therefore, wavefront manipulations such as focusing, deflection, and hologram can be achieved. To demonstrate this issue, we designed a metafilm‐based flat antenna working at 12 GHz with the similar structural profile in Figure 1d. The phase responses of the six‐leveled unit cells with different structural parameters (given in Table S1 in the Supporting Information) are calculated by the catenary model directly to uniformly cover 0–2π. Theoretically, any desired phase can be wrapped in this region without influence on the overall performance of the antenna. The diameter of our antenna is 800 mm, and the focal length is set as 400 mm as shown in Figure S7 (Supporting Information). In order to realize efficient focusing, the phase profile ϕ of our antenna follows

(4) $$\varphi (r)=-\frac{\omega }{c}\left(\sqrt{r^{2}+F^{2}}-F\right)$$

where ω, *c*, *r* and *F* are the angular frequency, vacuum light velocity, radial coordinate and focal length, respectively. The simulated focusing performance under normal incidence at 12 GHz is given in **Figure** **4**a, and the inset depicts the comparison of phase response between simulation and calculation. The fabrication process for the antenna is akin to the case for broadband absorber except for the employed metal (we employed aluminum in the antenna with σ = 3.56 × 107 S m^−1^). It should be mentioned that the minimal feature size of the antenna is only 50 µm in a 0.8 m diameter sample, which indicates our fabrication process can enable large‐scale and high‐precision fabrication simultaneously. To quantitatively evaluate the performance of this device, we simulated its gain at 12 GHz (as shown in Figure 4b,c). Owing to the limitations of the computation capability, a smaller antenna with 200 mm diameter and 100 mm focal length is employed in the simulation (details are given in Figure S8 in the Supporting Information). The gain of the small antenna is 22.8 dBi, which is very close to a traditional parabolic antenna with the same diameter (the corresponding gain is 24.6 dBi). As the area of the antenna is 16 times larger than the simulated one, the corresponding gain of the fabricated antenna is about 34.8 dBi. To employ this antenna for practical applications, we replaced the commercial antenna in the satellite television system with our metafilm‐based one to serve as the receiver for satellite signals. The operation satellite is ChinaSat 9 that emits circularly polarized signals in Ku band. The photo of the whole system is shown in Figure 4d. It can be seen from Movie S1 (Supporting Information) that the picture on the television is clear and smooth enough, which indicates this antenna can work properly in this system. The performance comparison of the metafilm‐based antenna with the commercial one is given in Figure S9 (Supporting Information) in terms of signal intensity and signal quality, which confirmed that the gain of the former one is above 30 dBi. In fact, the performance of this device can be further improved if the phase errors induced by near‐field coupling between adjacent unit cells are considered.^31^ Besides, benefiting from the recent works for broadband achromatic lens and real‐time tunable device, the operation bandwidth as well as the function of the antenna may be further expanded.^7^, ^8^, ^32^, ^33^ Apparently, this highly efficient metafilm‐based antenna has many promising applications especially in space and aviation communications.

## 4 Conclusion

In summary, we have demonstrated a systematic, general way to achieve efficient design of a certain kind of metasurface devices based on catenary electromagnetics. To simplify the case, we employed the CRR as the unit cell in our devices. As it has been proved that the frequency dispersions of more complicated structures such as double‐squared ring resonator^34^ and Jerusalem cross^35^ also follow the catenary model, the performance of the broadband absorber and metafilm‐based antenna can be further improved if these unit cells with more degrees of freedom are used. Besides, the design principle can be further extended to higher frequencies.^36^ We believe the direct linkage between the structure and its electromagnetic response based on catenary model will dramatically simplify the design process for metasurface‐based devices with decreasing time consumption and increasing robustness. With the rapid development of artificial intelligence (AI),^37^, ^38^ the optimization process can be further accelerated without extensive computation. Thus, the combination of catenary model with AI may open a new door for fast, robust, and efficient design.

As the field distributions as well as the frequency dispersions can be accurately described by catenary functions, the designed metafilm‐based devices show great advances versus their traditional counterparts owing to their lightweight properties and higher efficiencies. Besides, a fabrication process is proposed that enables high precision and batch production for these flexible substrate–based devices that paves the way for their future applications. The proposed method is a powerful tool to understand the inner physics (such as the subwavelength interference effects)^39^ of certain metasurfaces and is promising for inverse designs. The proposed devices have many potential applications in electromagnetic absorption, stealth technique, and space communications.

## 5 Experimental Section

*Numerical Simulation*: The numerical results in this paper for the unit cells were obtained by CST Microwave Studio based on the finite element method (the unit cell boundary was employed in the *x‐* and *y*‐directions and open in the *z*‐direction) and the results for the small antenna were based on the finite‐difference time‐domain method (open boundary (add space) is applied in every direction).

**Figure 5:**
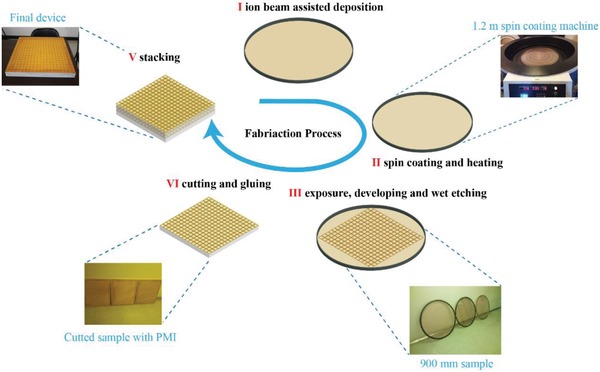
The fabrication process of our broadband absorber. The proposed technique can enable large‐scale fabrication with high precision on flexible substrates, which is promising in industrial applications.

*Device Fabrication*: The schematic diagram of the fabrication process is shown in **Figure** **5**. First, clean polymide films were stretched tightly by 0.9 m inner diameter carbon fiber holders. With the home‐made stretching machine, the flatness and uniformity of the film can be guaranteed for further processing. The deposition of nichrome was realized by ion beam‐assisted deposition at room temperature. As this process was performed at a relatively low temperature, it could avoid the thermal effect to the film. After that, the subsequent lithography processes were akin to their traditional counterparts but a set of equipment was developed, which could realize large‐scale spin coating, prebaking, exposure, developing, and wet etching. It should be noted that as the sample in this case was in meter scale, its exposure cannot be achieved directly. Thus, step exposure techniques were developed that can realize uniform step‐by‐step exposure. The maximum diameter for the sample can be as large as 1.2 m, which was far beyond its reported counterparts.

*Experimental Measurement*: The performance of the ultra‐broadband absorber was measured in an anechoic chamber with a vector network analyzer. In order to measure the reflection of this device, several pairs of high gain horn antennas working at different frequencies were employed.

## Conflict of Interest

The authors declare no conflict of interest.

## Supporting information

Supplementary

Supplementary
