# Supplementary material for: Catenary Electromagnetics for Ultra‐Broadband Lightweight Absorbers and Large‐Scale Flat Antennas
Source: Adv Sci (Weinh). 2019 Feb 1;6(7):1801691. doi: 10.1002/advs.201801691 (PMC6446607; doi:10.1002/advs.201801691)
Supplement: Supplementary file 1 — Supplementary [file ADVS-6-1801691-s002.pdf]

Copyright WILEY-VCH Verlag GmbH & Co. KGaA, 69469 Weinheim, Germany, 2018.

ADVANCED SCIENCE
Open Access

# Supporting Information

for *Adv. Sci.,* DOI: 10.1002/advs.201801691

# Catenary Electromagnetics for Ultra-Broadband Lightweight Absorbers and Large-Scale Flat Antennas

*Yijia Huang, Jun Luo, Mingbo Pu, Yinghui Guo, Zeyu Zhao, Xiaoliang Ma, Xiong Li, and Xiangang Luo**

**Catenary Electromagnetics for Ultrabroadband Lightweight Absorbers and Large-scale Flat Antennas**
**(Supporting Information)**

*Yijia Huang‡, Jun Luo‡, Mingbo Pu‡, Yinghui Guo, Zeyu Zhao, Xiaoliang Ma, Xiong Li, Xiangang Luo**

Dr. Yijia Huang, Dr. Jun Luo, Prof. Mingbo Pu, Dr. Yinghui Guo, Prof. Zeyu Zhao, Prof. Xiaoliang Ma, Prof. Xiong Li, Dr. Prof. Xiangang Luo
State Key Laboratory of Optical Technologies on Nano-Fabrication and Micro-Engineering, Institute of Optics and Electronics, Chinese Academy of Sciences, Chengdu 610209, China
E-mail: lxg@ioe.ac.cn
Dr. Yijia Huang
School of Optoelectronics, University of Chinese Academy of Sciences, Beijing 100049, China

## Part 1. Frequency Dispersions for Metallic Slits

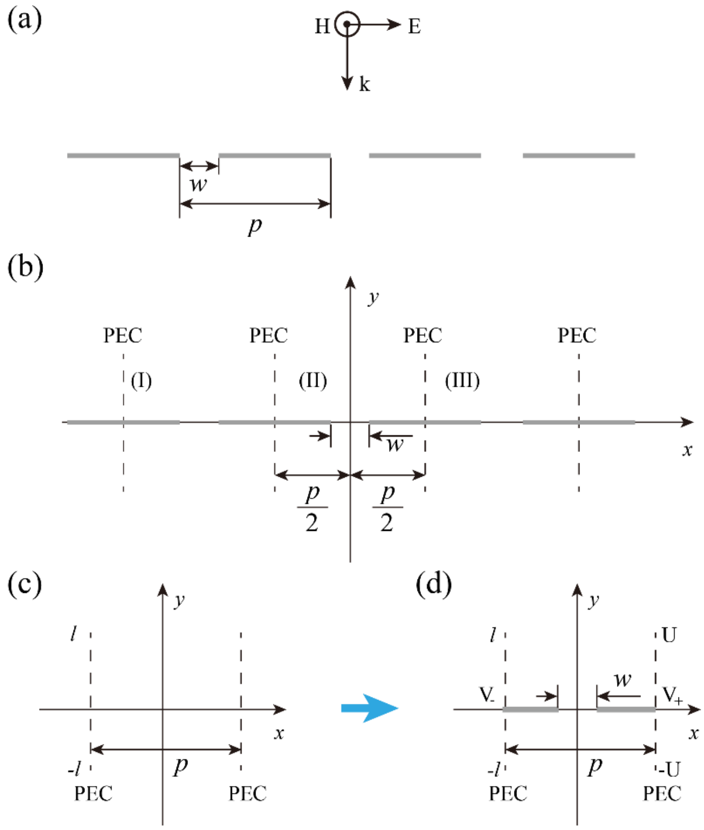


**Figure S1.** (a) The schematic of an array of metallic slits the same as the case in Figure 1a. (b) The modified treatment to the original structure that perfect electrical conducting (PEC) sheets are inserted in the central lines of the metallic films. (c) A single unit cell in (b) with metallic slits removed. (d) A single unit cell as shown in (b).

Figure S1(a) is the schematic of an array of metallic slits, which is the same as in Figure 1(a). A transverse magnetic plane wave is normally incident on this array. In order to deduce the explicit

functions to describe this subwavelength structure, a modified treatment is applied as shown in Figure S1(b) that PEC sheets are inserted in the metallic films. Owing to the symmetry of the structure, the insertion of PEC sheets parallel to *y* axis and perpendicular to the plane of the slits through the central lines of the films does not disturb the electric field. Therefore, the electric field of the whole array can be divided into numerous identical fields such as (I), (II) and (III) in Figure S1(b). If we can obtain the models for any of the small unit, we can apply them to other units as well. Thus, to simply the case, we choose unit (II) in Figure S1(b) as an example that the field is constrained from $x=-p/2$ to $x=p/2$.

As the electric field vibrates perpendicularly to the edges of the slits, the structure behaves as a purely capacitive device. Firstly, the background capacity of the modified structure is calculated by removing the metallic slits and maintaining the PEC sheets as shown in Figure S1(c). In this case, the plane wave propagates in the negative direction of *y* axis between two parallel PEC sheets. If we further constrain the space in *y* axis direction from $y=-l$ to $y=l$, the capacity of the structure in Figure 1(c) is

$$C'' = 2l\varepsilon_0 \tag{1}$$

where $\varepsilon_0$ is the vacuum permittivity. Then we return to the case as shown in Figure S1(d). The existence of the slits will distort the original electric field distributions and the influence can be described by the theory of Schwarz's conformal transformations in terms of equipotentials *V* and lines of force *U* at $(x,y)$ plane.[1]

$$\sin(V + iU) = \csc(\pi w / 2p)\sin[(x + iy)\pi / p] \tag{2}$$

By employing this function, the field distributions in *x*-*y* plane can be calculated. As the potential difference between two identical conductors is $V=\pi$, we assume that the potentials at $x=\pm p/2$ equal to $V_{\pm}=\pm\pi/2$. Then, at the boundary of $x=p/2$ Equation (2) can be calculated as

$$\sin(\frac{\pi}{2} + iU) = \csc(\frac{\pi w}{2p})\sin(\frac{\pi}{2} + i\frac{y\pi}{p}) \tag{3}$$

that can be simplified as

$$\cos(iU) = \csc(\frac{\pi w}{2p})\cos(i\frac{y\pi}{p}) \tag{4}$$

As $\cos(ia)=\cosh(a)$, *U* can be expressed as

$$U(y) = \pm\cosh^{-1}[\csc(\frac{\pi w}{2p})\cosh(\frac{y\pi}{p})] \tag{5}$$

Next, the total charge *Q* on the right PEC sheet can be calculated by the characteristic capacity $\kappa=p\varepsilon_0$ times *U* at the limits of the space from $y=-l$ to $y=l$.

$$Q = \kappa[U(l) - U(-l)] = 2\kappa U(l) \tag{6}$$

Thus, the capacity of the structure in Figure S1(d) can be calculated by

$$C' = \frac{Q}{V} = \frac{2\kappa}{\pi}\cosh^{-1}[\csc(\frac{\pi w}{2p})\cosh(\frac{\pi l}{p})] \tag{7}$$

Therefore, the difference in capacity is due to the existence of the slits and it can be deduced by

$$C = \lim_{l\to\infty}(C' - C'')$$

$$= \frac{2\kappa}{\pi}\ln\csc\frac{\pi w}{2p} \tag{8}$$

The corresponding impedance of the structure can be expressed by

$$Z_1 = 1/i\omega C = \frac{1}{i\frac{4p}{\lambda}\ln\csc(\frac{\pi w}{2p})} = \frac{1}{4iF} \tag{9}$$

where $\omega=2\pi c/\lambda$ is the angular frequency in the operation band, $c$ is the vacuum light velocity. This is Equation 1 in the main text and the vacuum impedance is omitted.

## Part 2. The Rigorous Deduction of Catenary Electric Field Distributions

As the Schwartz conformal transformation (Equation (3)) can quantitatively describe the field between the slits, the electric field intensity between the slit can be calculated by

$$E(x) = \frac{dV}{dx}\ (U=0,\ y=0) \tag{10}$$

With some mathematical deduction, Equation (11) can be written as

$$E(x) = \frac{\sqrt{2}\pi\cos(\frac{\pi x}{p})}{p\sqrt{\cos(\frac{2\pi x}{p}) - \cos(\frac{w\pi}{p})}} \tag{11}$$

This equation can be further expanded to a polynomial form as

$$E(x) = A + Bx^2 + Cx^4 + o(x^6) \tag{12}$$

where $A$, $B$ and $C$ are the coefficients in terms of $p$ and $w$ that can be obtained by Taylor expansion and $o$ is the high order term that can be omitted. Interestingly, the catenary function can be also expanded to the same mathematical form that $E(x)$ can be transformed to a typical catenary function

$$E(x) = a\cosh(bx) + c \tag{13}$$

where

$$a = \frac{2\pi\sin(\frac{\pi w}{2p})\cot^2(\frac{\pi w}{2p})}{p[17 + \cos(\frac{\pi w}{p})]} \tag{14}$$

$$b = \frac{\pi \cot(\frac{\pi w}{2p})}{p\sqrt{\frac{1+\cos(\frac{\pi w}{p})}{17+\cos(\frac{\pi w}{p})}}} \quad (15)$$

$$c = \frac{\pi}{p\sin(\frac{\pi w}{2p})} - \frac{2\pi \sin(\frac{\pi w}{2p})\cot^2(\frac{\pi w}{2p})}{p[17+\cos(\frac{\pi w}{p})]} \quad (16)$$

The above catenary function is a little complicated and can be simplified to an experiential expression when $w<<p$.

$$E(x) = \frac{1}{2\pi w}\cosh(\frac{7\cos(\frac{\pi w}{2p})}{w\cos(\frac{\pi w}{3p})}x) + \frac{\pi}{p\sin(\frac{\pi w}{2p})} - \frac{1}{2\pi w} \quad (17)$$

It can be found from Equation (11) that when $x$ approaches $\pm w/2$ the retrieved $E$ will reach infinity, which is impossible in reality. Thus, the above equations are valid except for the case at the two ends of the electric field. The comparisons of electric field intensity predicted by Equation (11), (13) and (17) are given in Figure S2. Apparently, both the rigorous and experiential catenary models matched the theoretical one quite well.

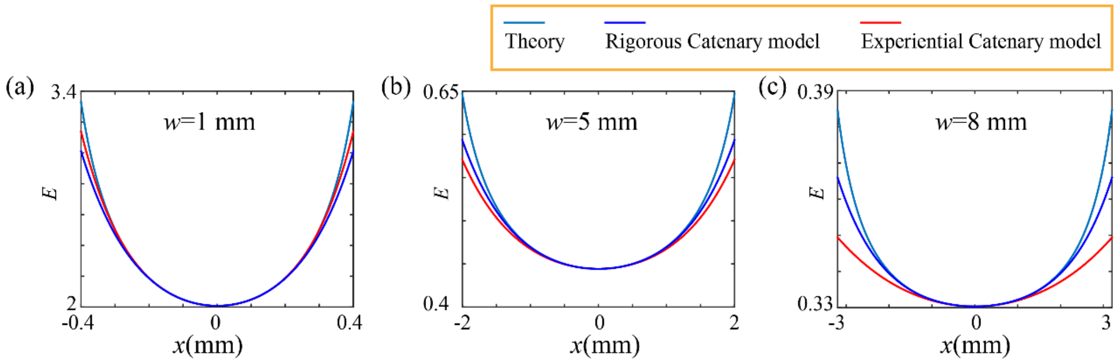


**Figure S2.** The electric field intensity distributions obtained by different models at various $w$ and fixed $p$=10 mm. Apparently, catenary models matched the theoretical one quite well.

In fact, the numerical simulations shown in Figure S3 even suggests that the catenary models can describe the field more accurately than Equation (11) when $w$ is much smaller than $p$.

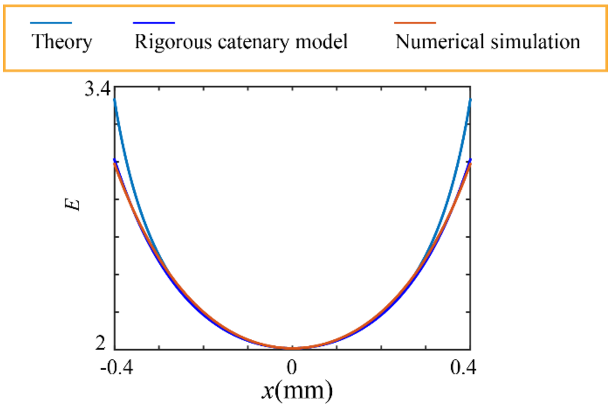


**Figure S3.** The electric field intensity distributions obtained by different methods at *w*=1 mm and *p*=10 mm. It can be seen that the catenary model matched the numerical simulation better than the theoretical one. The magnitude of the simulated result is normalized to the calculated ones.

## Part 3. Equivalent Circuit Theory for CRR

After solving the 1D case under transvers magnetic plane wave as shown above, the equivalent circuit model under the transvers electric (TE) wave can be easily obtained by applying the Babinet's principle that the product of the impedance for complementary slits equal to one-quarter the square of the vacuum impedance.[2] Thus, the model in this case is

$$Z_2 = iF \tag{18}$$

It should be noted that *w* is the length of a metallic film in this case instead of the width of the slit.

As we have deduced the equivalent circuit models for metallic slits under both polarizations, the corresponding expressions for CRR can be easily obtained. Considering the capacitance *C* of the CRR, the corresponding susceptance can be drawn by

$$B_C = \omega C = 4\frac{p-g}{p}F(p,g) \tag{19}$$

The susceptance is reduced by a factor (*p*-*g*)/*p* is owing to the surface not being continuous. The reactance of the inductance *L* can be expressed by

$$X_L = \omega L = \frac{p-g}{p}F(p,2s) \tag{20}$$

Again the reactance is reduced by (*p*-*g*)/*p* and the factor 2 before *s* is because there are two inductive slits in a unit cell. The effective impedance $Z_{eff}$ of a given CRR, thus, can be calculated by

$$Z_{eff} = R + i(X_L - \frac{1}{B_C})Z_0 \tag{21}$$

where *R* is the resistance of the effective sheet.

$$R = R_s\frac{p^2}{2ls} \tag{22}$$

## Part 4. Transmission Line Theory

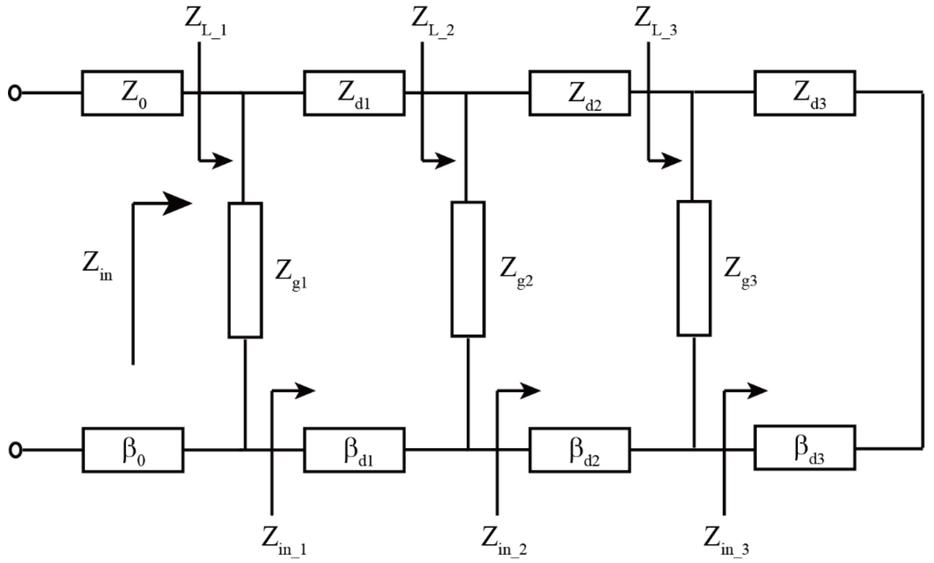


**Figure S4.** The transmission line model of our BA. $Z_{gi}$, $Z_{di}$ ($i$=1,2 and 3) are the effective impedance of the metasurface and the impedance of dielectric. $\beta_{di}$ ($i$=1,2 and 3) are the propagation constant in the dielectric. $Z_0$, $Z_{\mathrm{in}}$ and $Z_{\mathrm{L}}$ are the characteristic impedance of free space, input impedance and output impedance of the corresponding layer.

According to the transmission line theory, the parameters in Figure S4 follow:

$$Z_{L\_i} = \frac{Z_{gi} Z_{in\_i}}{Z_{gi} + Z_{in\_i}} \tag{23}$$

$$Z_{in\_i+1} = Z_{di} \frac{Z_{L\_i} + jZ_{d(i+1)} \tan(\beta_d d_{(i+1)})}{Z_{d(i+1)} + jZ_{L\_i} \tan(\beta_d d_{(i+1)})} \tag{24}$$

where $\beta_d$ and $Z_d$ can be expressed as

$$\beta_d = \frac{\omega}{c}\sqrt{\varepsilon_d} \tag{25}$$

$$Z_d = \frac{Z_0}{\sqrt{\varepsilon_d}} \tag{26}$$

Using the iterative algorithm, we can recursively derive the input impedance of the entire equivalent circuit model from the bottom layer. The reflection coefficient can be calculated by the following equation.

$$r = \frac{Z_{in} - Z_0}{Z_{in} + Z_0} \tag{27}$$

Then, we can calculate the absorption of the proposed BA by $A=1-|r|^2$.

**Part 5. Gain calculation for the parabolic antenna**

The gain $G$ of the parabolic antenna is obtained by the following empirical equation

$$G(dBi) = 10\lg(4.5 \times (D/\lambda_0)^2) \quad (28)$$

where $D$ is the diameter of the antenna and $\lambda_0$ is the working wavelength.

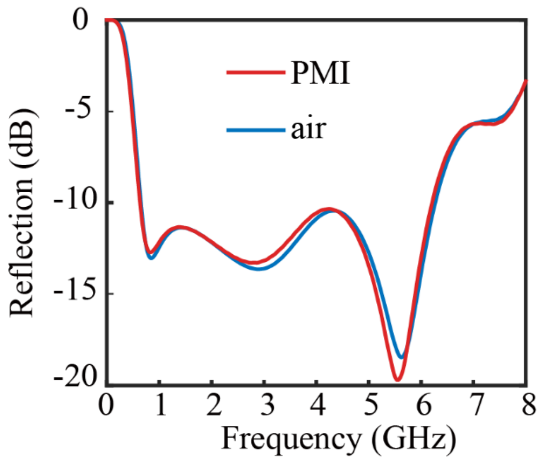


**Figure S5.** The comparison of reflection between PMI and air as the dielectric layer. As the permittivity of PMI is very close to air, the simulated results are nearly the same.

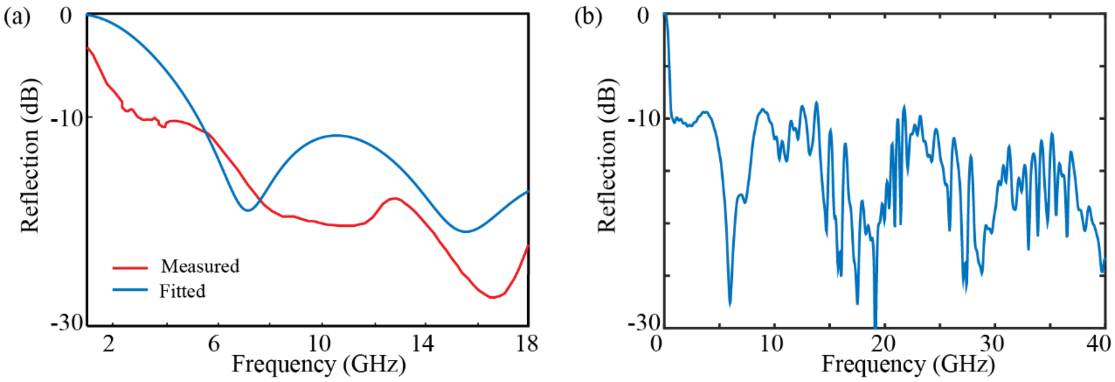


**Figure S6**. (a) The measured (red) and fitted (blue) reflectance of the honeycomb absorbing material. It should be noted that the seller can only present the reflectance of the material from 2-18 GHz and the permittivity as well as permeability is unknown. The fitted result is obtained by assuming the permittivity of the material equals 0.8+0.64i and permeability equals 1. (b) The simulated reflectance for the device in Figure 3(c).

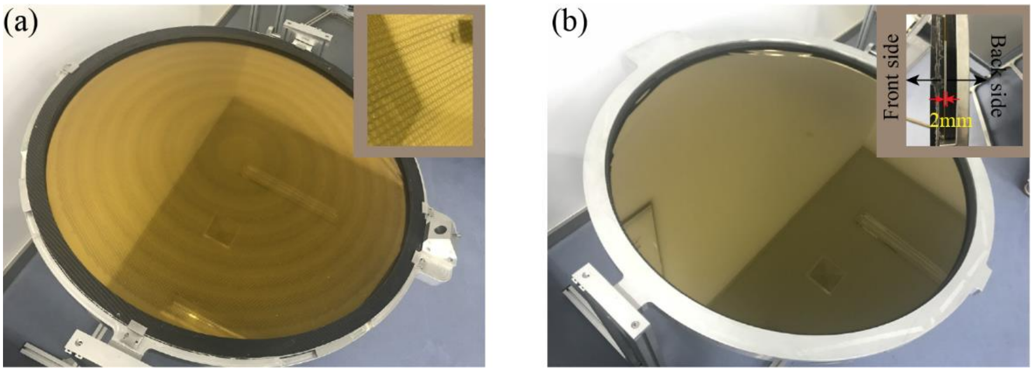


**Figure S7.** The images of our flat antenna. (a) The front side of the antenna with 800 mm diameter is composed of subwavelength unit cells. The inset is the zoom-in picture of the flat antenna. (b) The back side of the antenna behaves as a film mirror. As shown in the inset, we separate the two films by several small metal plate with the thickness of 2 mm at the edge of the carbon fiber holder. In that case, the 2 mm air gap between the two films can be guaranteed.

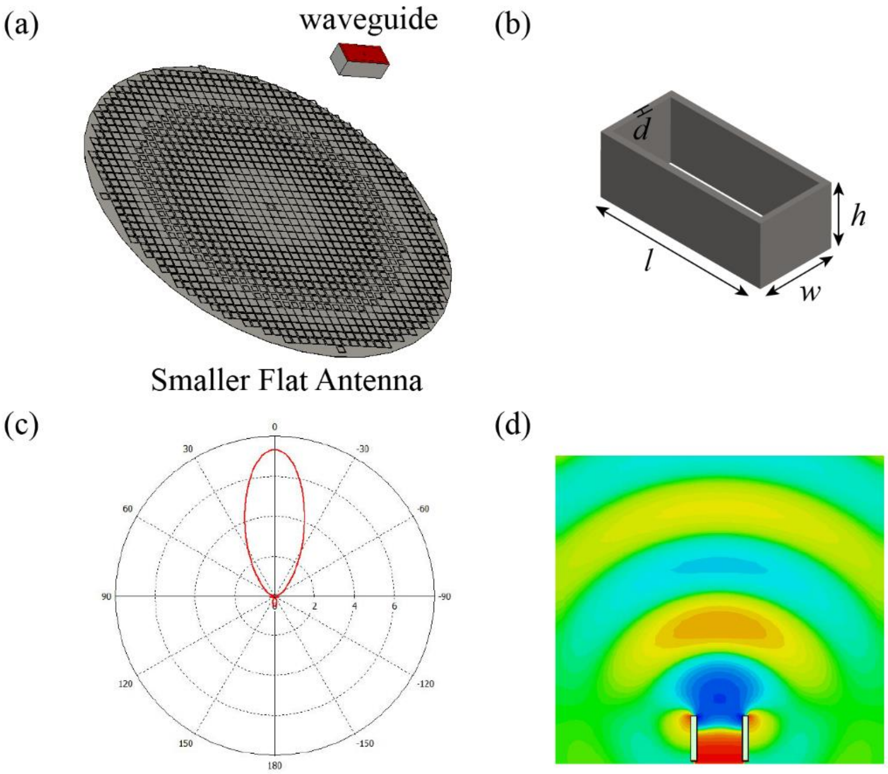


**Figure S8.** (a) The schematic of the simulation for Figure 5(b) and 5(c). The waveguide is placed 100 mm above the central line of the 200-mm-diameter antenna. (b) The geometry of the waveguide with *l*=25.4 mm, *w*=12.7 mm, *h*=10 mm and *d*=1.27mm. (c) The farfield pattern of the waveguide at 12 GHz that shows good directivity. (d) The electric field distribution of the waveguide that indicates this waveguide can be treated as a quasi-spherical wave source.

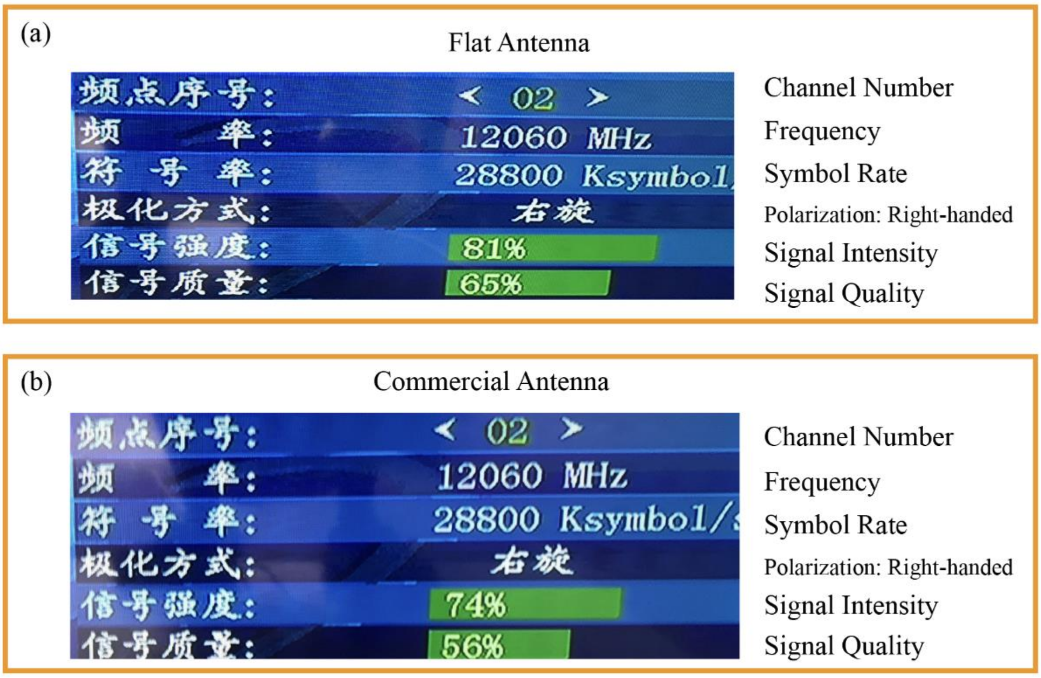


**Figure S9.** The performance comparison of our flat antenna and the commercial one. It can be seen that the signal intensity as well as the signal quality obtained by our flat antenna is larger than that of the commercial one. As the gain of the latter one is about 30 dBi according to its guide book, the corresponding gain of our antenna is above 30 dBi.

**Table 1** Calculated phase response of the six-leveled unit cells in the film antenna with *p*=5 mm, *d*=2 mm, *t*=100 nm

| Number | 1 | 2 | 3 | 4 | 5 | 6 |
|---|---|---|---|---|---|---|
| *g* (mm) | 1.5 | 0.8 | 0.6 | 0.5 | 0.35 | 0.05 |
| *s* (mm) | 0.2 | 0.2 | 0.25 | 0.2 | 0.2 | 0.1 |
| Relative Phase (deg) | 0 | 60 | 120 | 180 | 240 | 300 |

**Reference**

[1] W. R. Smythe, *Static and Dynamic Electricity*, McGraw-Hill, New York, **1939**.
[2] H. G. Booker, *J. I.E.E.* **1946**, 93, Part IIIA, 620.
